# Effect of Tilt Angle and Pin Depth on Dissimilar Friction Stir Lap Welded Joints of Aluminum and Steel Alloys

**DOI:** 10.3390/ma12233901

**Published:** 2019-11-26

**Authors:** Veerendra Chitturi, Srinivasa Rao Pedapati, Mokhtar Awang

**Affiliations:** Department of Mechanical Engineering, Universiti Teknologi PETRONAS, Seri Iskandar 32610, Malaysia; srinivasa.pedapati@utp.edu.my (S.R.P.); mokhtar_awang@utp.edu.my (M.A.)

**Keywords:** dissimilar materials, friction stir lap welding, pin depth, tilt angle, tool rotational speed, welding speed

## Abstract

Automobile, aerospace, and shipbuilding industries are looking for lightweight materials for cost effective manufacturing which demands the welding of dissimilar alloy materials. In this study, the effect of tool rotational speed, welding speed, tilt angle, and pin depth on the weld joint were investigated. Aluminum 5052 and 304 stainless-steel alloys were joined by friction stir welding in a lap configuration. The design of the experiments was based on Taguchi’s orthogonal array for conducting the experiments with four factors and three levels for each factor. The microstructural analysis showed tunnel defects, micro voids, and cracks which formed with 0° and 1.5° tilt angles. The defects were eliminated when the tilt angle increased to 2.5° and a mixed stir zone was formed with intermetallic compounds. The presence of the intermetallic compounds increased with the increase in tilt angle and pin depth which further resulted in obtaining a defect-free weld. Hooks were formed on either side of the weld zone creating a mechanical link for the joint. A Vickers hardness value of HV 635.46 was achieved in the mixed stir zone with 1000 rpm, 20 mm/min, and 4.2 mm pin depth with a tilt angle of 2.5°, which increased by three times compared to the hardness of SS 304 steel. The maximum shear strength achieved with 800 rpm, 40 mm/min, and a 4.3 mm pin depth with a tilt angle of 2.5° was 3.18 kN.

## 1. Introduction

The joining of two different materials which are completely different in their mechanical, physical, and chemical properties is always a challenge. Aluminum and steel are very difficult to weld because of the large difference in their melting temperatures. Friction stir welding (FSW), which was introduced in 1991 at The Welding Institute, has the advantage over fusion welding techniques in terms of joining dissimilar materials. Friction stir welding depends entirely on how much heat is produced during the process, an outcome of the friction between the rotation tool and the fixed plates. If the heat generated is too high, the aluminum may reach its melting point which will not result in a sound joint; if the heat generated is not enough to stir the steel into aluminum, it will result in a joint with many defects. Thus, process parameters, such as tool rotational speed, welding speed, pin depth, and tilt angle, play a crucial role in deciding the weld quality [1,2].

Friction stir lap welding (FSLW) of aluminum and steel is usually carried with the aluminum on top and the steel on the bottom so that the heat generated will be enough for plastic deformation of the materials to form a strong joint. Elrefaey et al. [3] welded commercially pure aluminum and low carbon steel plates with different ranges in process parameters to investigate the joint strength and concluded that a minimal difference in the pin depth will have a large impact on the joint strength. Lap joints between AA 5083 and SS 304 of 3 mm thickness were produced by Kimapong et al. [4] with a pin depth of 3–3.3 mm and found that increasing the rotational speed decreased the joint strength and vice versa with traverse speed and more intermetallic compounds forming with increased pin depth. Chen et al. [5] welded three different types of steel with AC4C alloy and found that zinc-coated steel had better fracture strength when compared with brushed and mirror-finished steel which was nearly 97.7% of the chosen steel. The effect of the cylindrical pin on 1.5 mm AA6181-T4 and HC340LA joints was studied by Coelho et al. [6] where 0.2 µm thick intermetallic compounds were formed and the weld had 73% joint strength of the aluminum base material. When Movahedi et al. [7] welded 3 mm thick AA 5083 and St-12 with different tool rotational speeds and welding speeds, they found that the joint strength increased with decreasing welding speed, and the microstructure revealed the presence of intermetallic compounds. The effect of tool rotational speed and traverse speed on 6063 aluminum and 1 mm thickness zinc-coated steel with a pin depth of 3.1 mm was investigated by Das et al. [8] and achieved 58.6% failure load of the base aluminum alloy. Studies on FSLW of 1060 aluminum alloy and 1Cr18Ni9Ti steel were investigated by Wei et al. [9] which revealed that the joint strength increased with the increase in pin depth when the tilt angle was maintained at 0°. The maximum shear load of 7500N was achieved by Wan et al. [10] when 6082-T6 aluminum and Q235A steel were friction stir lap welded with a tilt angle of 2.5° and resulted in defect-free weld. Various process parameters were used by Nguyen et al. [11] to weld DP800 and AA6351 sheets, and they noticed that the weld joints with high intermetallic thickness had a higher joint strength.

Not only do the process parameters and plunge depth effect the joint properties, but the pin geometry along with pin material will also have an impact on the joint strength. A new cutting-edge pin geometry was introduced by Xiong et al. [12]; the pin was made with YG8 cemented carbide rotary burrs and inserted into an H13 tool holder to weld 1100 aluminum alloy and 1Cr18Ni9Ti stainless steel sheets which yielded a shear strength of 89.7 MPa, which is stronger than that of the aluminum base alloy. Shamsujjoha et al [13] used W-Re-HfC pin tools to weld 1018 stainless steel and 6061 aluminum alloy and found that the longer pin tool achieved the highest joint strength which was 58% of the base aluminum alloy. Pseudo steady-heat transfer analysis conducted by Das et al. [14] proved that the presence of Al_13_Fe_4_ intermetallic compound (IMC) increases with the increase in rotational speed and decrease in welding speed which results in almost a 70% joint strength of steel. Further, the study on intermetallic compounds formed between the weld joints of 6061 aluminum and high-strength interstitial free steel proved that thermo-dynamically stable IMC’s are formed, confirming that diffusion plays a major role in joint strength [15]. The microstructure of the lap welded joints of AA 6061-T6 and low carbon steels revealed that the thickness of the IMC layer decreases with the increase in welding speed which, in turn, will affect the joint strength [16]. Annealing was used to increase the joint strength of 3 mm 5083 aluminum alloy and 1 mm St-12 steel sheet by Movahedi et al. [17] when at 300 °C and 350 °C. The joint strength increased with the increase in annealing time, but the joint strength decreased as the temperature was increased to 400 °C. Haghshenas et al. [18] found that the joint strength achieved by diffusion bonding of 5754 aluminum alloy with coated high-strength steels increased with the decrease in welding speed and increase in pin depth. Fatigue crack propagation and fracture toughness of friction stir lap welded 3003 aluminum and SUS304 were examined by Hidehito et al. [19] who noticed that fracture toughness depends on crack propagation. A novel technique called self-riveting FSLW was developed by Huang et al. [20] to join a 6082-T6 aluminum alloy to QSTE340TM steel, and they proved that the riveting technique in FSLW will improve the fracture load of the joint. 

It is evident from the abovementioned studies [1,2,3,4,5,6,7,8,9,10,11,12,13,14,15,16,17,18,19,20] that the selection of process parameters plays a significant role in determining the robustness of the weld. The objective of this study was to investigate the effect of tilt angle and pin depth on the welded joint. The tool rotational speed ranges used were from 800 rpm to 1200 rpm and the welding speed ranges used were from 20 mm/min to 40 mm/min. The pin depths chosen for the experimental procedure were between 4.1 mm and 4.3 mm with tilt angles ranging from 0° to 2.5°.

## 2. Experimental Methodology

The base metals chosen for this study were aluminum 5052 alloy with the dimensions 200 mm × 100 mm × 4 mm and 304 stainless steel alloy with the dimensions 200 mm × 100 mm × 2 mm. The chemical compositions of the base metals are shown in Table 1.

**Figure 1:**
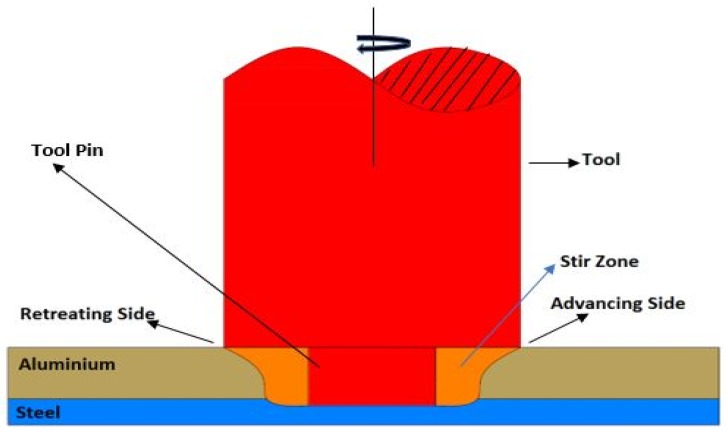
Schematic view of friction stir lap welding.

The base metals are welded in lap joint configuration with CNC FSW machine with a constant inflow of argon gas to cool the tool from overheating. The schematic diagram of FSLW is shown in Figure 1. Tool material was made with Tungsten–Rhenium (W–Re), with a shoulder diameter of 20 mm, cylindrical shaped pin diameter of 5 mm with a pin length of 4.1 mm. Based on the preliminary results, the process parameters chosen for the design of experiments were tool rotational speed (TRS), welding speed (WS), pin depth (PD), and tilt angle (TA). The ranges of these parameters are shown in Table 2. The design of the experiments included four factors, each having three levels, and the optimized number of experiments were chosen according to the Taguchi L9 orthogonal array as shown in Table 3. The experiments were conducted using a load force of 7.5 kN.

**Figure 2:**
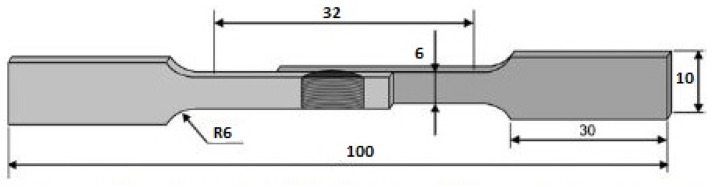
Dimensions of the tensile shear test specimen (mm).

The welded joints were cut according to ASTM E8 (sub-size) standards perpendicularly to the welding direction using wire-cut electrical discharge machining (EDM, Mitsubishi, Tokyo, Japan) for tensile shear testing. The dimensions of the tensile shear test specimen are shown in Figure 2. The tensile shear tests were carried out on a 100 kN universal testing machine at a speed rate of 3 mm/min. The hardness of the welded joints was determined with a Vickers hardness testing machine (Leco LM 247AT, St. Joseph, MI, USA) by applying a load of 500 gf with a dwell time of 15 s. The hardness values were taken in the mixed stirred zone along the cross-section. Samples for microstructural analysis were cut in 20 mm × 20 mm size with wirecut EDM. Keller’s reagent was used to etch the 5052 aluminum side and Nital was used to etch the SS 304 side. Microstructure analysis was conducted using scanning electron microscopy (SEM, Phenom Pro X, Eindhoven, Netherlands).

## 3. Results and Discussion

### 3.1. Macrostructure of the Lap Joints

**Figure 3:**
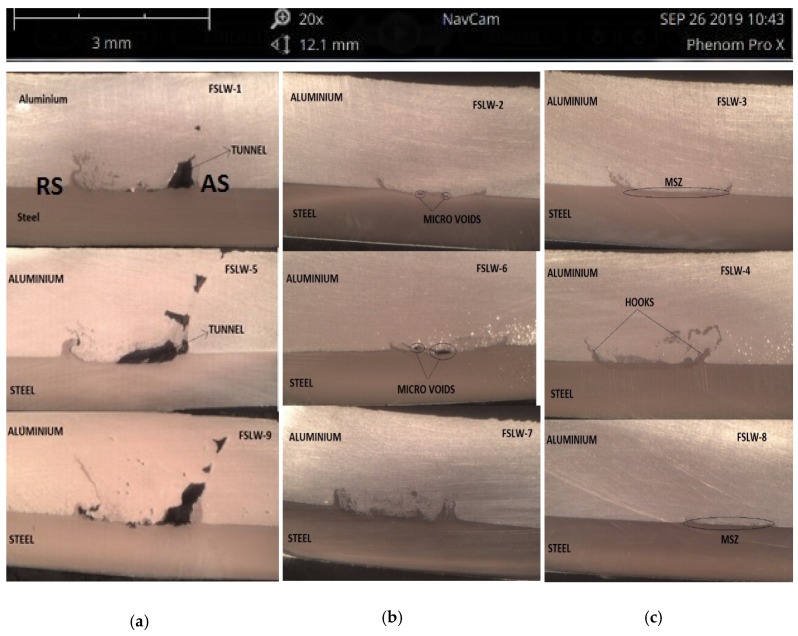
Macrostructure of the specimens with different tilt angles: (**a**) 0° Tilt Angle, (**b**) 1.5° Tilt Angle, (**c**) 2.5° Tilt Angle.

Macrostructures of the welded samples will give a good perception of the welded zone. The experiments were carried out with a 0° tilt angle, and the formation of a tunnel defect can be observed in Figure 3a. This was the result of not enough stirring of material and heat input, because the aluminum was thrown out of the mixed stir zone while stirring. The tunnel defect size increased with the increase in the depth and resulted in more flash formation during the welding. When the tilt angle was increased to 1.5 degrees with the same rotational and traverse speed and pin depth, the tunnel defect was reduced, but some micro voids were formed (Figure 3b) in FSLW-2 and FSLW-6 where the pin depth was 4.1 mm (0.1 penetration mm in steel) and 4.2 mm (0.2 penetration mm in steel). The micro voids were reduced to micro-cracks when the pin depth increased to 4.3 mm (0.3 penetration mm in steel) in FSLW-7. The macrostructure of the dissimilar FSLW joint is shown in Figure 3c, where a sound and defect-free joint was obtained. In the nugget zone, the material was extruded from the advancing side towards the retreating side of the weld, forming a mechanical link between the aluminum and steel plates.

### 3.2. Microstructure of the Lap Joints

**Figure 4:**
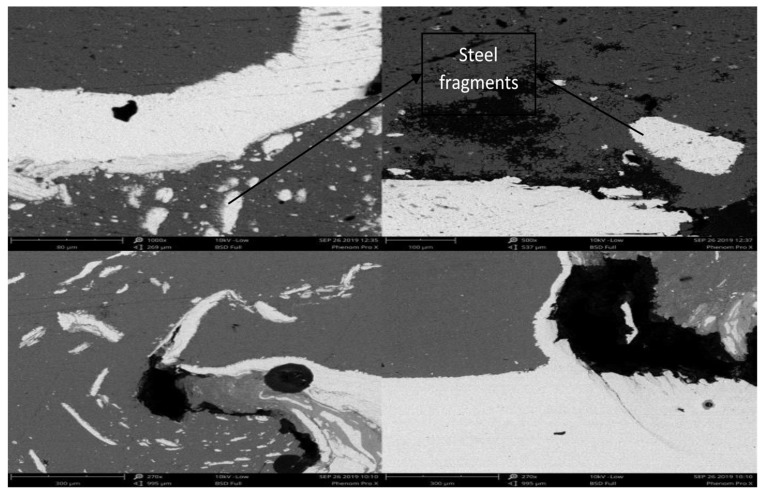
SEM images of the mixed stir zone with a 0° tilt angle.

Microstructures of the welded zone were obtained using a scanning electron microscope. As a result of the tunnel defect shown in Figure 4, different sizes of scattered steel fragments were found in the mixed stir zone. The uneven distribution of steel fragments into aluminum increased with the increase in tool rotational speed. Due to the movement of the tool in the welding direction, the steel particles from the surface of the steel were dragged along the welding direction which is the reason for the uneven scattering of the steel fragments. The tunnel defect was observed on the advancing side whereas the stir zone on the retreating side was good and without any defects. 

**Figure 5:**
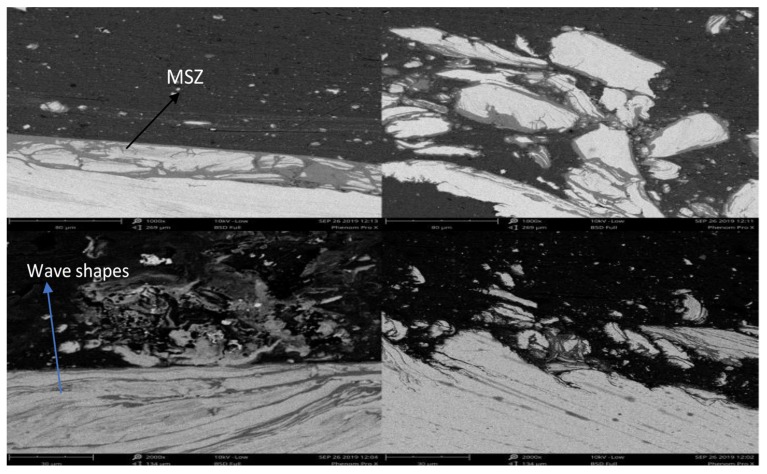
SEM images of the mixed stir zone with a 1.5° tilt angle.

Increasing the tilt angle to 1.5° reduced the tunnel defect, but the formation of micro voids and root cracks can be observed. The uneven distribution of steel and aluminum fragments is observed in Figure 5 in the mixed stir zone (MSZ). The rotation of the tool was in the clockwise direction, and by increasing the tilt angle, the escaping of material from the stir zone was reduced, and because of the pin depth, a mixed stir zone was formed consisting of aluminum and steel fragments alongside the intermetallic compounds. A wave-like streamline was detected which is evidence that the mixture of both materials is occurring. However, the flash formed was less when compared to the 0° tilt angle, because the stirred material was pushed inside instead of escaping from the stirred zone.

**Figure 6:**
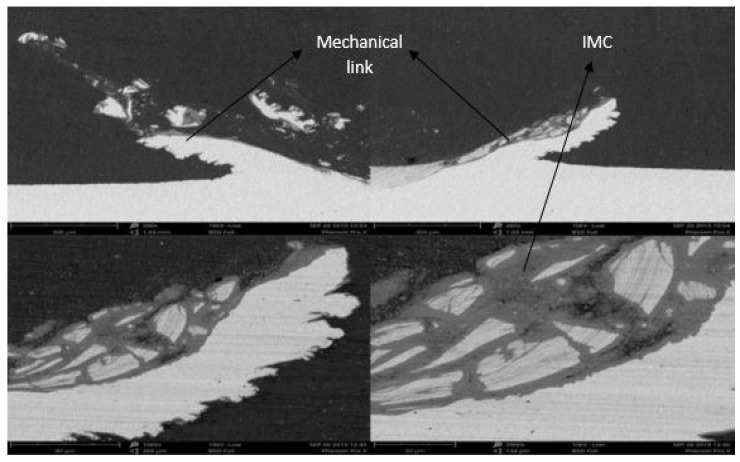
SEM images of the mixed stir zone with a 2.5° tilt angle.

Further increasing the tilt angle to 2.5°, a sound weld was obtained with proper mixing of aluminum and steel and without any defects as can be seen in Figure 6. The small voids which were seen with the 1.5° tilt angle were also eliminated. The increase in tilt angle and pin depth eliminated the uneven distribution of fragments in the stir zone. There was a hook-like mechanical link formed on either side of the stir zone as shown in Figure 6. The formation of the hook depends on the depth of the pin. Even here, the intermetallic compounds can be perceived in the mixed stir zone (MSZ) of the weld. The mechanical link which was formed on either side of the weld zone holds a crucial role in deciding the strength of the joint. The hook formed with FSLW-8 was less when compared to FSLW-3 and FSLW-4, and, as a result, the maximum tensile shear strength of 3.18 kN and 3.13 kN was achieved with FSLW-3 and FSLW-4, respectively.

### 3.3. Mechanical Properties of the Lap Joint

Tensile shear test results showed that a maximum of 3.18 kN shear strength can be achieved with a tool rotational speed of 800 rpm, welding speed of 40 mm/min with a pin depth of 4.2 mm when the angle was tilted to 2.5 degrees, and the least, 0.64 kN, was obtained with a TRS of 1000 rpm, WS of 30 mm/min, with a pin depth of 4.3 when the angle was not tilted. It is evident from the results that tensile shear strength mostly depends on the tilt angle and pin depth rather than tool rotational speed and welding speed. The top three tensile shear test results were obtained when the angle was tilted to 2.5 degrees, the least three were obtained when there was no tilt in the angle, and the medium results were obtained when the angle was tilted to 1.5 degrees.

**Figure 7:**
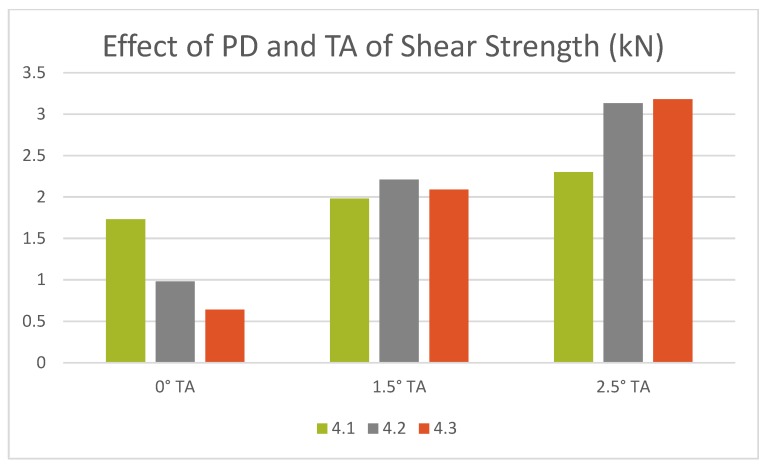
Effect of tilt angle on shear strength at various pin depths.

The effect of pin depth and tilt angle on tensile shear strength is shown in Figure 7. At 0° tilt angle, the shear strength decreased with the increase in pin depth, and, at a 2.5° tilt angle, it increased with the increase in pin depth. This pattern shows that the increase in tilt angle can stir and hold the material together in the mixed stir zone such that the tunnel defects, which were formed at 0°, are eliminated. But, when at 1.5°, the pin depth did not show much variation in the joint strength.

**Figure 8:**
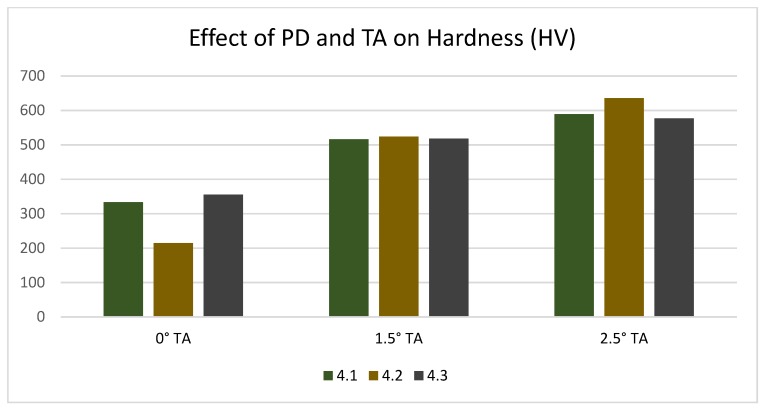
Effect of tilt angle on the hardness at various pin depths.

The Vickers hardness results confirm that a maximum of 635.46 HV was obtained when the tool rotational speed was 1000 rpm, welding speed was 20 mm/min, the pin depth was 4.2, and the tilt angle 2.5°. The hardness results also followed the same pattern as the tensile shear test results, where the highest hardness was obtained when the tilt angle was 2.5° and the least when the tilt angle was 0°. The pin depth variation did not show much effect on the hardness like the tilt angle. As shown in Table 4, the hardness values with different pin depths at the same tilt angles were almost near to one another. The effect of the pin depth and tilt angle on hardness is shown in Figure 8. According to Basariya et al. [21], high hardness values can be obtained when intermetallic compounds are formed.

### 3.4. Signal-to-Noise Ratio Analysis

The obtained tensile shear strength and hardness values were further analyzed using Taguchi’s signal-to-noise (S/N) ratios to obtain the influence of factors on the responses. The approach for the S/N ratio in this study was “the larger the better” in order to obtain the maximum response. The calculated S/N ratios for the criteria for “the larger the better” and mean responses are shown in Table 5.

**Figure 9:**
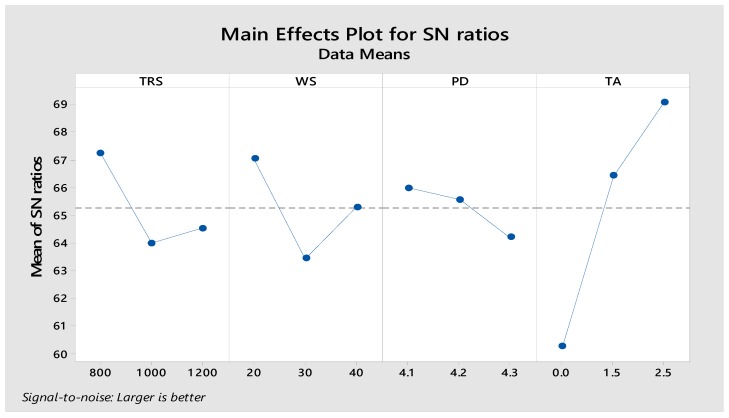
The signal-to-noise ratio plot for different process parameters.

The output of S/N ratios are plotted in Figure 9. According to “the larger the better” criteria, it is revealed from the plot that the maximum tensile strength and hardness can be achieved with a tool rotational speed of 800 rpm, welding speed of 20mm/min, 4.1 mm pin depth, and a tilt angle of 2.5° where the tilt angle is the most influential factor ranked at 1 followed by welding speed, tool rotational speed, and pin depth.

## 4. Conclusions

In this study, friction stir lap welding of 5052 aluminum and 304 stainless-steel alloys were successfully achieved without any defects, and the following conclusions were drawn from the results.

1. Microstructural analysis revealed that a defect-free joint can be achieved with a tilt angle of 2.5° if the mixed stir zone is visible with respect to pin depth. Micro voids are formed if the tilt angle is reduced to 1.5° with steel fragments scattering in the mixed stir zone, and tunnel defects are clearly visible on the advancing side of the stir zone and uneven distribution of steel particles on the retreating side when the tilt angle is further reduced to 0°.
2. Tensile shear test results showed that a maximum strength of 3.18 N was achieved with 800 rpm, 40 mm/min, and a pin depth of 4.3 mm when the angle was tilted to 2.5° due to the fact of a good mixed stir zone with intermetallic compounds.
3. Eliminating the micro voids and tunnel defects achieved a maximum hardness of HV 635.36 with 1000 rpm, 20 mm/min, and a pin depth of 4.2 mm when the angle was tilted to 2.5° and when a defect-free weld was obtained.
4. From the microstructural analysis and tensile shear strength result, it is evident that a better joint strength is achieved with a tilt angle of 2.5° and a pin depth of 4.3 mm.

## Figures and Tables

**Table 1:** Composition of 304 stainless steel and 5052 aluminum.

**SS 304 Elements**	**Fe**	**Cr**	**Ni**	**Mn**	**N**	**S**	**C**	**Si**	**P**
Percentage (wt.%)	Bal	18	8	2	0.1	0.03	0.08	0.75	0.045
**AA 5052 Elements**	**Al**	**Cr**	**Mg**	**Mn**	**Fe**	**Cu**	**Zn**	**Si**	**Others**
Percentage (wt.%)	Bal	0.15–0.35	2.2–2.8	0.1	0.4	0.1	0.1	0.25	0.15

**Table 2:** Process parameters and their levels.

Parameter	Level 1	Level 2	Level 3
Tool Rotational Speed (rpm)	800	1000	1200
Welding Speed (mm/min)	20	30	40
Pin Depth (mm)	4.1	4.2	4.3
Tilt Angle (Degree)	0	1.5	2.5

**Table 3:** Taguchi L9 orthogonal array.

Run	TRS (rpm)	WS (mm/min)	PD (mm)	TA (degree)
FSLW-1	800	20	4.1	0
FSLW-2	800	30	4.2	1.5
FSLW-3	800	40	4.3	2.5
FSLW-4	1000	20	4.2	2.5
FSLW-5	1000	30	4.3	0
FSLW-6	1000	40	4.1	1.5
FSLW-7	1200	20	4.3	1.5
FSLW-8	1200	30	4.1	2.5
FSLW-9	1200	40	4.2	0

**Table 4:** Hardness and tensile shear strength values of the specimens.

Run	TRS (rpm)	WS (mm/min)	PD (mm)	Tilt Angle (degree)	Hardness (HV)	Shear Strength (kN)
FSLW-1	800	20	4.1	0	333.32	1.73
FSLW-2	800	30	4.2	1.5	524.02	2.21
FSLW-3	800	40	4.3	2.5	576.9	3.18
FSLW-4	1000	20	4.2	2.5	635.46	3.13
FSLW-5	1000	30	4.3	0	355.42	0.64
FSLW-6	1000	40	4.1	1.5	516.42	1.98
FSLW-7	1200	20	4.3	1.5	517.9	2.09
FSLW-8	1200	30	4.1	2.5	588.86	2.30
FSLW-9	1200	40	4.2	0	214.84	0.98

**Table 5:** Signal-to-noise ratios of the experiments.

Level	Tool Rotational Speed	Welding Speed	Pin Depth	Tilt Angle
**1**	67.25	67.04	65.99	60.25
**2**	63.99	63.43	65.57	66.43
**3**	64.52	65.29	64.21	69.08
**Delta**	3.25	3.61	1.78	8.83
**Rank**	3	2	4	1
